# The Great Still-Usion: Unmasking Adult-Onset Still’s Disease Masquerading As Upper Respiratory Tract Infection

**DOI:** 10.7759/cureus.35880

**Published:** 2023-03-07

**Authors:** Zainab Qudsiya, Donica L Baker

**Affiliations:** 1 Internal Medicine, St. Luke's Hospital, Chesterfield, USA; 2 Rheumatology, St. Luke's Hospital, Chesterfield, USA

**Keywords:** still’ s disease, upper respiratory tract infection, major hyperferritinemia, hyperferritinemia, fever of unkown, adult onset still's disease (aosd), adult-onset still’s disease

## Abstract

Adult-onset Still's disease (AOSD) is a systemic inflammatory disorder of unknown etiology that presents with high-grade fever, arthritis, evanescent rash, and multiorgan involvement. It is a rare disorder and is a diagnosis of exclusion. AOSD is often misdiagnosed initially as viral exanthems or upper respiratory tract infections leading to a delay in diagnosis. Management includes non-steroidal anti-inflammatory drugs (NSAIDs), glucocorticoids, and conventional or biologic disease-modifying antirheumatic drugs (DMARDs). We report a case of a 53-year-old female with prolonged fever, sore throat, arthralgia, and rash. She was initially presumed to have infectious pharyngitis but did not respond to antimicrobial therapy. After extensive evaluation that excluded infectious, malignant, and other rheumatological etiologies, she was noted to satisfy multiple Yamaguchi criteria and was subsequently diagnosed with AOSD. Glucocorticoids and biologic DMARDs were initiated, leading to improved clinical manifestations and a decline in inflammatory markers.

## Introduction

Adult-onset Still's disease (AOSD) is a rare systemic inflammatory disorder that occurs with an incidence of 0.16-0.4 per 100,000 adults. It has a bimodal age distribution that peaks between the ages of 15-25 years and 36-45 years and is proposed to occur due to immune dysfunction [1,2]. This condition most commonly presents with fever, rash, arthralgia, sore throat, lymphadenopathy, and hepatosplenomegaly. Due to overlapping clinical manifestations and lack of a specific diagnostic test, AOSD is commonly mistaken initially for acute viral syndromes, malignancy, or other autoimmune disorders leading to inappropriate therapy. This case highlights the importance of considering AOSD as a differential diagnosis of fever of unknown origin once other etiologies have been excluded, especially in the presence of serum hyperferritinemia.

## Case presentation

A 53-year-old female with a history of depression and hyperlipidemia presented with a two-week history of high-grade fever ranging from 38.3 to 39.4 C, chills, fatigue, sore throat, myalgia, and polyarthralgia involving both wrists, elbows, and ankle joints. This was accompanied by unintentional weight loss of 6 lb over two weeks and a non-pruritic rash occurring with fever spikes. She denied a recent history of sick contacts, recent travel, tick bites, or a family history of autoimmune conditions. On admission, vitals signs were temperature 38.3 C, heart rate 73 beats/minute, blood pressure 102/55 mm Hg, respiratory rate 15/minute, and oxygen saturation of 98% on room air. The examination was notable for a coalescent erythematous macular circular rash over the thighs and forearms, left axillary lymphadenopathy, and mild swelling and tenderness of bilateral wrist and ankle joints. The head and neck exam was unremarkable, with no evidence of pharyngeal exudates or follicles. Otherwise, the systemic exam was unrevealing. She was initially evaluated at urgent care, where tests for rapid streptococcal antigen and COVID-19 were negative. She was treated with oral amoxicillin for a week. Due to persistent symptoms and lack of response to antimicrobials, she was admitted for evaluation of a fever of unknown origin. 

Investigations were notable for neutrophilic leukocytosis (WBC count 18,600/microliter, neutrophils 91%), normocytic anemia (hemoglobin 10.6 gram/deciliter), elevated inflammatory markers (erythrocyte sedimentation rate [ESR] 60 millimeter/hour, C-reactive protein 21.3 milligram/deciliter), marked hyperferritinemia (serum ferritin 5140 nanogram/milliliter), and transaminitis (aspartate transaminase 52 unit/liter) (Tables 1-3). Initial infectious workup for other bacterial (tuberculosis, brucellosis, Q fever) and viral etiologies (COVID-19, respiratory 22 plex, HIV, Ebstein-Barr virus [EBV], parvovirus) was negative (Table 4). In view of persistently negative blood cultures, lack of cardiac murmur, embolic phenomenon, or vegetations on transthoracic echo, infective endocarditis was ruled out. Next, the possibility of malignancy was considered. CT of the chest, abdomen, and pelvis was negative for malignancy, incidentally showing mild splenomegaly, trace pericardial effusion, and bilateral pulmonary atelectasis. Age-appropriate cancer screening was up to date. Her most recent pap smear was normal. A colonoscopy done two years prior revealed benign tubular adenomas that had been resected. Her most recent mammogram showed breast tissue with scattered areas of fibro glandular density (category B) and left supraclavicular lymphadenopathy. Subsequent excisional biopsy of the left supraclavicular lymph node did not reveal any evidence of malignancy or lymphoma. Apart from the positive anti-nuclear antibody (ANA) (1:160), the workup for rheumatoid arthritis, systemic lupus erythematosus (SLE), and other systemic autoinflammatory disorders was negative (Table 5). No articular destruction was noted on wrist or hand X-rays. During admission, the patient endorsed a new-onset headache and jaw claudication. In view of these symptoms and elevated ESR, bilateral temporal artery biopsies were conducted that were negative for temporal arteritis. 

**Table 1:** Hematology g/dL: Gram/deciliter; fl: Femtoliter; pg: Picograms; U/L: Unit/liter; pg/ml: Picogram/milliliter; ng/ml: Nanogram/milliliter.

Hematology	Lab values on admission	Reference range
WBC	18,600 /microliter	4,300-10,000/microliter
Hemoglobin (Hb)	10.6 gram/deciliter (g/dL)	11.8-14.8 g/dL
Mean Corpuscular Volume (MCV)	82.1 femtoliters (fl)	82-95 fl
Mean Corpuscular Hemoglobin (MCH)	29.6 picograms (pg)	27.2-32.6 pg
Platelet	181x 106/microliter	140-350 x 106/microliter
Lactate Dehydrogenase (LDH)	950 units/liter (U/L)	313-618 U/L
Reticulocyte	1%	0.4-2.2 %
Vitamin B12	907 picogram/milliliter (pg/mL)	239-931 pg/mL
Folate	10.9 nanogram/milliliter (ng/mL)	>2.8 ng/mL

**Table 2:** Biochemistry. meq/L: Milliequivalent/liter; mg/dL: Miligram/deciliter; U/L: Units/liter; miU/L: Milli international units/liter.

Biochemistry	Lab values on admission	Reference range
Sodium (Na)	137 milliequivalents/liter (mEq/L)	137-145 mEq/L
Potassium (K)	3.6 mEq/L	3.5-4.9 mEq/L
Carbon Dioxide (CO2)	22 mEq/L	22-30 mEq/L
Chloride (Cl)	108 mEq/L	98-107 mEq/L
Blood urea nitrogen (BUN)	18 milligram/deciliter (mg/dl)	7-17 mg/dl
Creatinine	0.6 mg/dl	0.5-1 mg/dl
Uric acid	2 mg/dL	2.5-6.2 mg/dl
Creatine Kinase (CK)	58 unit/ liter (U/L)	30-135 U/L
Thyroid-stimulating hormone (TSH)	1.15 milli international units/liter ( mIU/L)	0.47-4.68 mIU/L
Aspartate transaminase (AST)	52 U/L	14-36 U/L
Alanine transaminase (ALT)	15 U/L	<35 U/L
Bilirubin	0.7 mg/dL	0.2-1.3 mg/dL
Cholesterol	169 mg/dL	<200 mg/dL
Direct High Density Lipoprotein (HDL)	36 mg/dL	>=41 mg/dL
Low density lipoprotein (LDL)	114 mg/dL	=<99 mg/dL
Triglyceride	259 mg/dL	<=149 mg/dL

**Table 3:** Inflammatory markers on admission. mm/hr: Millimeter/hour; mg/dL: Milligram/deciliter; ng/ml: Nanogram/milliliter.

Inflammatory markers	Lab values on admission	Reference range
Erythrocyte sedimentation rate (ESR)	60 millimeter/hour (mm/hr)	0-30 mm/hr
C-reactive protein (CRP)	21.3 milligram/deciliter (mg/dL)	0.0-0.9 (mg/dL)
Ferritin	5140 nanogram/milliliter (ng/mL)	11-264 ng/ml

**Table 4:** Infectious diseases evaluation.

Infectious Diseases Evaluation	
Urine analysis	Leucocyte Esterase (LE) negative, nitrite negative, trace protein
Blood cultures	Done thrice, no growth
QuantiFERON-TB Gold Plus	Negative
Human immunodeficiency virus Antigen/Antibody (HIV-1/2 Ag/Ab) screen	Negative
Streptococcus Group A screen, culture	Negative
SARS-CoV-2 RNA	Done thrice, negative.
Respiratory 22 plex Polymerase Chain Reaction (PCR)	Negative for Adenovirus, Coronavirus HKU1, NL63, 229E, OC43, Metapneumovirus, Rhinovirus/Enterovirus, Influenza A, B, Parainfluenza 1-4, RSV, Bordetella pertussis, parapertussis, Chlamydia pneumoniae, Mycoplasma pneumoniae, SARS-CoV-2
Epstein-Barr virus immunoglobulin M (EBV Ig M)	Negative
Infectious Mononucleosis heterophile antibodies	Negative
Cytomegalovirus immunoglobulin M (CMV IgM)	Negative
Parvovirus B19 immunoglobulin G, immunoglobulin M	Negative
Q Fever IgG, Ig M	Negative
Brucella abortus IgG, IgM	Negative

**Table 5:** Autoimmune evaluation. mg/dL: Milligram/deciliter.

Autoimmune workup	
Antinuclear Antibody (ANA)	1:160, homogenous pattern
Double-stranded DNA antibody (dsDNA)	Negative
Ribonucleoprotein antibody (RNP)	Negative
Anti-Smith antibody (Sm)	Negative
Sjögren’s Syndrome A and B Antibodies SSA, SSB	Negative
Scleroderma Antibody (SCL-70)	Negative
Jo-1 Antibody	Negative
Rheumatoid Factor (RF) Screen	Negative
Cyclic Citrullinated Peptide Immunoglobulin G (CCP IgG)	Negative
Complement 3 (C3)	176 milligram/deciliter (mg/dL) (Reference 82-167 mg/dL)
Complement 4 (C4)	29 mg/dL (Reference 12-38 mg/dL)

**Figure 1:**
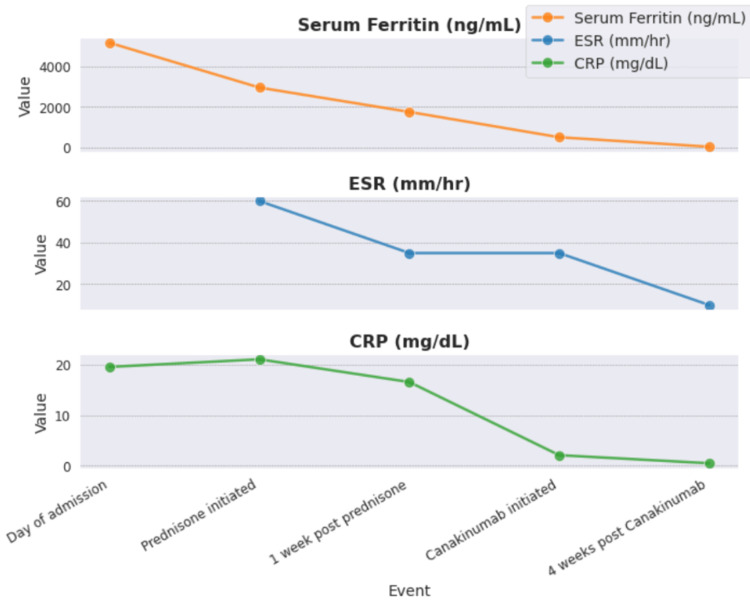
Trend of inflammatory markers over the course of the treatment. ESR: Erythrocyte sedimentation rate; CRP: C-reactive protein; ng/ml: Nanogram/milliliter; mm/hr: Millimeter/hour; mg/dL: Milligram/deciliter.

After extensive negative workup for malignancy and infectious diseases, the patient's clinical presentation was noted to satisfy multiple Yamaguchi criteria, and she was diagnosed with AOSD. A tapered regimen of oral prednisone starting from 20 mg was initiated, which led to clinical improvement and a decline in inflammatory markers. She followed an intermittent course and had a systemic flare of symptoms (fever, sore throat, rash) five weeks after initiation of prednisone. She was eventually transitioned to injectable canakinumab (anti-interleukin-1 [IL-1]) every 4- 8 weeks, which led to improvement (Figure 1). 

## Discussion

The etiology of AOSD is unclear, although genetic, infectious, and immune triggers have been proposed. Mechanisms of immune dysregulation include neutrophil and macrophage activation, increased T helper Th1/Th2 ratio, natural killer cell dysfunction, elevated T helper 17 activity, and increase in pro-inflammatory cytokines including interleukins IL1B, IL6, IL18, tumor necrosis factor (TNF-α) [3-5]. The leading clinical manifestations include fever (95%), arthralgia or arthritis (95%), and rash (77%). Fever classically follows a quotidian (daily recurrent fever) or double quotidian pattern (two spikes per day) with or without defervescence between spikes. However, our patient did not demonstrate a clear quotidian or double quotidian pattern. As seen in our patient, joint involvement may occur in the form of mild transient oligo-arthritis involving the knees, wrists, ankles, elbows, interphalangeal joints, and shoulders. This can evolve into severe destructive polyarthritis over time. The typical rash of AOSD is a salmon-colored evanescent maculopapular eruption that occurs with fever spikes and disappears with defervescence, most commonly involving the trunk and extremities but can also affect the palms, soles, and face. However, atypical rashes have also been described [6]. Severe myalgia may occur during fever spikes with mild to no elevation of creatine kinase and aldolase. Non-suppurative pharyngitis due to cricothyroid perichondritis or aseptic pharyngitis occurs in 53% of cases. Other findings include pleuritis, pericarditis, weight loss, mild, tender symmetrical lymphadenopathy, and mild splenomegaly. Abdominal pain can occur due to lymphadenitis, acute pancreatitis, and aseptic peritonitis. Elevated acute phase reactants, leukocytosis, normocytic anemia, and transaminitis may be seen. Rheumatoid factor (RF) and ANA are negative in most cases. However, 7% of patients may be positive for ANA which does not exclude the diagnosis [7,8]. Serum ferritin levels are significantly higher in AOSD than other rheumatic conditions and correlate with disease activity. Marked hyperferritinemia >1000 nanogram/milliliter (ng/mL) (more than five times the upper limit of normal) is more suggestive of AOSD. Low glycosylated ferritin (<20%) is a more specific diagnostic test. However, its utility is limited due to the lack of widespread availability. Nonerosive narrowing of the carpometacarpal and intercarpal joint spaces of the wrist with ankylosis is a characteristic radiologic finding in AOSD. AOSD is a diagnosis of exclusion based on clinical and laboratory features in the absence of other mimicking conditions. Several diagnostic criteria have been proposed for AOSD. The Yamaguchi criteria have high discriminative power with a sensitivity and specificity of 96% and 98%, respectively, and are often used to establish the diagnosis (Table 6) [9,10]. The diagnosis of AOSD requires the presence of five Yamaguchi criteria, with at least two being major diagnostic criteria. Our patient appropriately underwent a comprehensive evaluation that ruled out infectious causes, including acute viral syndromes, infective endocarditis, systemic autoimmune conditions, and malignancy. Thereafter, she was found to satisfy three major and four minor criteria that led to the final diagnosis of AOSD. AOSD can follow a monocyclic course with a single systemic episode, intermittent/polycyclic, or chronic disease patterns. Systemic signs and symptoms predominate in monocyclic and polycyclic courses, while articular involvement is more common in chronic disease.

**Table 6:** Yamaguchi diagnostic criteria for AOSD. Diagnosis of AOSD requires the presence of five criteria, with at least two being major diagnostic criteria. AOSD: Adult-onset Still's disease

Major Criteria	Minor Criteria
1. Fever of at least 39 °C for at least 1 week	1. Sore throat
2. Arthralgia or arthritis for at least 2 weeks	2. Lymphadenopathy
3. Non-pruritic salmon-colored rash on the trunk/extremities	3. Hepatomegaly or Splenomegaly
4. Granulocytic leukocytosis (10,000/mL or greater)	4. Abnormal liver function tests
	5. Negative in tests for Rheumatoid Factor (RF) and Anti Nuclear Antibody (ANA)

Macrophage activation syndrome (MAS) is a severe and life-threatening complication of AOSD, which may present similarly to an AOSD flare with fever, splenomegaly, and increased ferritin and can lead to multiorgan failure. It is characterized by the presence of activated macrophages in the bone marrow that phagocytose hematopoietic cells. However, MAS is associated with leukopenia, thrombocytopenia, hypofibrinogenemia, hypertriglyceridemia, and elevated lactate dehydrogenase, differentiating it from AOSD. 

The goals of therapy are to control inflammation and prevent end-organ damage. Management depends on disease severity and risk of macrophage activation syndrome. Treatment options include NSAIDS, glucocorticoids, conventional DMARDs (methotrexate, leflunomide), and biologic agents (anakinra, canakinumab, tocilizumab, infliximab, etanercept). Mild disease (fever, rash, mild joint involvement) is initially managed with inflammatory doses of NSAIDs. Lack of response to NSAIDS and moderate to severe diseases (serositis, severe polyarthritis, high-grade fever, and internal organ involvement) requires remission induction with glucocorticoids with steroid-sparing conventional/biologic DMARDs. Initial prednisone doses of 0.5-1 mg/kg/day are continued until control of symptoms and lab markers are noted, followed by tapering. A total of 70% of patients respond to glucocorticoids. DMARDs are added if steroids cannot be tapered to ≤10 mg daily of prednisone after a month of therapy. IV pulse steroid therapy is indicated for life-threatening diseases (disseminated intravascular coagulation [DIC], macrophage activation syndrome [MAS], cardiac tamponade, and hepatic involvement). Biologic DMARDs are indicated in severe or refractory disease resistant to glucocorticoids and second-line conventional DMARDs. These biologic agents target the blockade of pro-inflammatory cytokines such as IL-1 (anakinra-IL-1R antagonist), (canakinumab, rilonacept- anti-IL-1β monoclonal antibody), IL-6 (tocilizumab) and TNF (etanercept, infliximab).

## Conclusions

Due to its rarity, overlapping clinical manifestations, and lack of specific serological markers, diagnosing AOSD is often challenging. This case emphasizes the importance of considering AOSD in the differential diagnosis of fever of unknown origin and persistent pharyngitis. In such cases, the presence of markedly elevated serum ferritin and the exclusion of other autoimmune, infectious, and malignant etiologies should prompt physicians to consider this diagnosis.
